# Sauropod dinosaur tracks from the Purbeck Group (Early Cretaceous) of Spyway Quarry, Dorset, UK

**DOI:** 10.1098/rsos.240583

**Published:** 2024-07-03

**Authors:** Richard J. Butler, Kirsty M. Edgar, Lewis Haller, Luke E. Meade, Harry T. Jones, Oliver Hill, Sam Scriven, Christopher Reedman

**Affiliations:** ^1^ School of Geography, Earth and Environmental Sciences, University of Birmingham, Birmingham B15 2TT, UK; ^2^ Jurassic Coast Trust, Brooklands Farm, Forston, Dorchester DT2 7AA, UK

**Keywords:** sauropod, Cretaceous, Spyway Quarry, Dorset, dinosaur, track

## Abstract

Dinosaur tracks have a long history of discovery and study in the UK, but track sites for sauropodomorph dinosaurs—the group that included the giant, graviportal herbivorous sauropods—are comparatively rare. Here, we provide a description of a sauropod dinosaur track site at Spyway Quarry in Dorset, southern England. The tracks at Spyway were discovered in the late 1990s and occur in the Stair Hole Member of the Durlston Formation in the Purbeck Limestone Group, of earliest Cretaceous age. More than 130 individual tracks of large sauropod dinosaurs are present at the site, but they are generally poorly preserved and do not form clear trackways, although it is likely that they represent multiple individuals. They provide further evidence for sauropods living in or passing through coastal lagoonal environments. Although poorly preserved, Spyway represents the largest *in situ* dinosaur track site currently accessible within the Purbeck Group, with considerable potential for further discoveries through ongoing quarrying in the surrounding area.

## 1. Introduction

Dinosaur tracks have a long history of study in the UK, beginning in the mid-nineteenth century (e.g. [1]) but with new sites still being documented (e.g. [2–4]) as part of a wider ‘dinosaur track renaissance’ (e.g. [5]). Close to 300 distinct dinosaur track occurrences have been documented from the UK, each representing a track morphotype at a particular horizon and locality [6], although *in situ* dinosaur trackways (as opposed to isolated and often *ex situ* tracks) are rare in the UK, with approximately 14 key localities currently accessible [6].

Sauropodomorph dinosaurs, the group that includes giant, graviportal herbivorous sauropod dinosaurs such as *Brachiosaurus* and *Diplodocus*, originated in the Middle–Late Triassic and survived until the end of the Cretaceous [7], but based on the body fossil record, they were most diverse (at least in the Northern Hemisphere) during the Middle and Late Jurassic (e.g. [8]). A diversity decline of sauropodomorphs is proposed to have occurred during the Early Cretaceous, although interpretations of diversity changes through the Jurassic to Cretaceous transition are complicated by sampling biases [8,9]. The body fossil record of sauropodomorphs in the UK is relatively poor with limited taxonomic diversity (e.g. [10–12]), probably as a result of the limited availability of fossiliferous terrestrial sediments in the intervals of peak sauropodomorph diversity during the Jurassic [6]. Likewise, sauropodomorph tracks are rare in the UK, comprising only 16% of occurrences. The UK dinosaur track record is instead dominated by tridactyl (three-toed) tracks produced by theropods or ornithopods, comprising approximately 75% of all known occurrences [6].

Key UK localities preserving sauropodomorph tracks *in situ* today include Late Triassic sites at Bendrick Rock and Penarth in South Wales [4,13], Middle Jurassic sites at Ardley Quarry in Oxfordshire [14–16], Rubha nam Barthairean and Cairidh Ghlumaig in Scotland [2,3] and the Early Cretaceous of Spyway Quarry, near Acton in Dorset [17].

The fossil footprints at Spyway were first identified in 1997 by local quarryman Kevin Keates when removing the Upper Freestone of the Durlston Formation of the Purbeck Limestone Group. The top surface of the underlying Bottom Freestone was covered with numerous large, shallow bowl-shaped depressions. These were subsequently identified by Keates, together with another local quarryman, Trev Haysom, as potential dinosaur footprints. The land on which the tracks occur is owned by the National Trust and was leased to Keates Quarries Ltd. The tracksite was known for many years as Keates Quarry, but the name was later changed to Spyway Quarry after the opening of the site for public access in 2016 to minimize confusion with the name of the quarry firm. Following the discovery, quarrying temporarily ceased for an initial scientific investigation commissioned by the National Trust and led by Joanna Wright [18]. Following this work, the tracks were covered using an anti-weed, geosynthetic fabric covered with crushed stone (K Keates 2021, personal communication) to facilitate quarrying of the immediately surrounding area before being re-exposed in 2013.

While a comprehensive internal National Trust report was developed documenting the site as initially exposed [18], only a brief half-page report (without figures) was ever formally published [17]. Ferraby & Powesland [19] described a publicly available photogrammetric model of the site produced in 2014 and Edgar *et al*. [20] described the impacts on the site of it being opened to public access and the elements. However, aside from these geoconservation-focused contributions, the site has been largely invisible in the scientific literature, receiving only brief occasional mentions [21,22]. This, combined with changes in the exposed quarry surface through time yielding multiple additional tracks [20], technological advances in the methods used to record and report tracks (e.g. [5]), and advances in understanding of the UK dinosaur track record [6] mean that the time is ripe for a revised description of the site. Thus, this study provides an up-to-date description of the Spyway Quarry dinosaur tracks, detailing the nature of the individual tracks and current inferences for the nature of the trackmakers and their behaviour.

## 2. Geological setting

**Figure 1.**
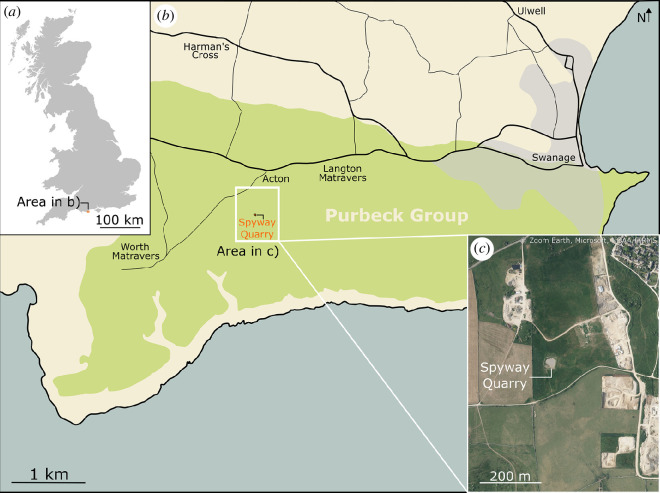
Maps showing (*a*) the general location of Spyway Quarry (orange dot) along the Dorset coast, UK. (*b*) The area surrounding Spyway Quarry and the local extent of the Purbeck Group (green-shaded area). (*c*) A satellite image of Spyway Quarry and the surrounding active Keates and Lewis Quarries (image taken from Zoom Earth). Figure re-used from Edgar *et al*. [20].

**Figure 2.**
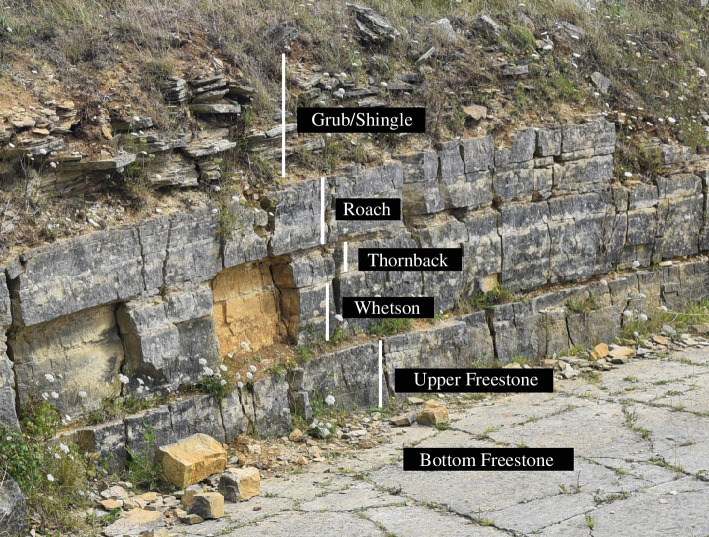
Annotated photograph showing the exposed part of the Stair Hole Member of the Durlston Formation on the eastern side of Spyway Quarry, with terms for individual units used by the local quarry workers marked. The sauropod tracks occur on the top of the Bottom Freestone.

Spyway Quarry is located between Langton Matravers and Worth Matravers, East Dorset (50.60216° N, 2.02020° W; figure 1), and is locally designated as a regionally important geological site (RIGS; under its previous name of ‘Keates Quarry’) by Dorset’s Important Geological Sites Group. The dinosaur tracks occur on the top surface of a bed locally known as the Bottom Freestone, part of the Freestone Vein within the Stair Hole Member (which incorporates the frequently cited Intermarine Member of Clements [23] within which the Freestone occurs) of the Durlston Formation in the Purbeck Limestone Group [24]. The Stair Hole Member comprises interbedded shelly limestones (biomicrites and biosparites) and mudstones deposited in a marginal marine environment. The Stair Hole Member is dated to the Berriasian of the Early Cretaceous [24]. Approximately 6 m section of the overlying beds of the Stair Hole Member is exposed on the eastern side of the quarry above the track surface (figure 2).

A second discovery of sauropod tracks on the top surface of the Bottom Freestone was made at Lewis Quarries, approximately 200 m east of Spyway Quarry, in 2018 [25].

The Freestone Vein includes coarse bioclastic limestones within the Stair Hole Member that have been extensively quarried for building stone for the past few hundred years. The Bottom Freestone is approximately 30 cm thick locally with a pale yellow/white colour on a fresh surface and contains bivalve shells (less than 1 cm) with some larger oyster fragments and ostracods. These sediments are thought to have been deposited on the edge of a freshwater lagoon separated from the open sea [26]. The overlying Upper Freestone, which covers the footprint horizon, is a similar shelly limestone, but shells are more chaotically arranged and inferred to be rapidly deposited, possibly during a storm event [18].

Dinosaur tracks have been discovered at multiple stratigraphic levels within the Lulworth and Durlston formations of the Purbeck Limestone Group [17,27,28]. The greatest abundance of tracks comes from the Intermarine beds within the Stair Hole Member [17,27].

## 3. Methods

**Figure 3.**
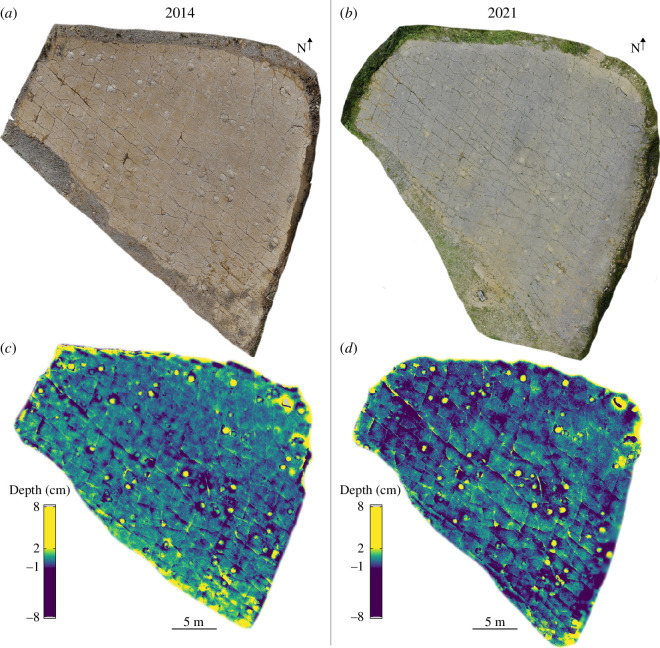
Textured photogrammetric models of Spyway Quarry surface from 2014 (*a*) and 2021 (*b*). Height maps of flattened photogrammetric models of the Spyway Quarry surface from (*c*) 2014 and (*d*) 2021, detailing prominent tracks, cracks and other reliefs. Minor colour differences generally reflect slight differences in flattening or photogrammetric model accuracy, rather than a change over time. Some detail around quarry edges has been lost during flattening. Figure re-used from Edgar *et al*. [20].

Spyway Quarry was studied during multiple visits between summer 2020 and early 2023, including a detailed examination of the track-bearing surface, sedimentary logging in nearby quarries and study of the remaining Lewis Quarries tracks, which are currently stored at Keates Quarries Ltd. We generated a new photogrammetric model of the whole of Spyway Quarry in August 2021 (figure 3). Our methods largely follow those used by Ferraby & Powesland [19] to generate the publicly available April 2014 model (https://skfb.ly/oDvzt) and are described in detail in Edgar *et al*. [20]. The 2021 Spyway model is publicly available on Figshare (https://figshare.com/articles/media/3D_model-2021_Spyway_Quarry_Dorset_UK/21256107). In this study, we present new photogrammetric models of 109 individual tracks (or pairs of adjacent tracks) from Spyway and two blocks containing tracks from Lewis Quarries using Agisoft Metashape Standard (v. 1.7.5) and Professional (v. 1.7.4) editions, following methods in Edgar *et al*. [20]. Photos were taken on a Nikon D750 digital SLR camera with an 18−55  mm f/3.5−5.6G lens. Individual photogrammetric models are available at https://doi.org/10.5281/zenodo.11509629. Height maps for selected tracks were generated in the software CloudCompare (v. 2.12.3).

**Figure 4.**
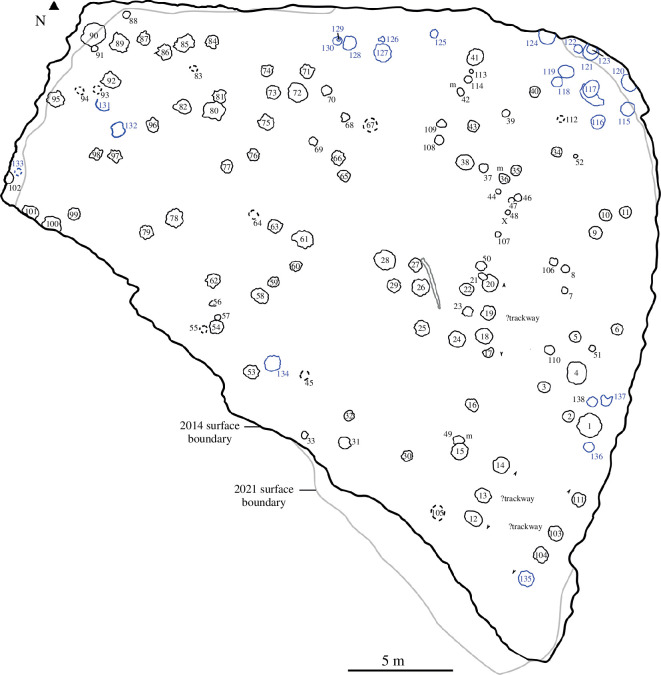
Schematic line map of Spyway Quarry showing dinosaur track and quarry outline between 1998 and 2021. Tracks are numbered 1–138 following Edgar *et al.* [6]. Poorly preserved tracks or those that are no longer readily apparent are marked by dashed lines. Changes in the quarry surface extent between 2014 and 2021 are shown by the black and grey lines, respectively. Three possible trackways are defined between the arrows. Minor modifications are made to the map presented in Edgar *et al*. [6]: the position of a second trace fossil interpreted as formed by wood is marked by a grey cross in addition to the long linear feature in the centre of the map marked in grey, and the tracks that we tentatively describe as manus are indicated by ‘m’. Of the two tracks labelled as track 49 in Edgar *et al*. [6], an error propagated from Wright *et al*. [18], the northernmost has been identified based on the description in Wright *et al*. [18] and relabelled as track 44. Tracks 115−138 identified in Edgar *et al*. [6] are now shown in blue.

To compare the Spyway track surface across the 2014 and 2021 three-dimensional models with the original line map by Wright *et al*. [18], we created a line map highlighting track positions and other significant features on the quarry surface (figure 4). Without flattening the quarry surface, screenshots of the quarry surface from both three-dimensional models were captured from the default view in Metashape and imported into Inkscape software. In Inkscape, a new layer was generated, and tracks were drawn free-hand onto this layer. Subsequently, the track layers from 2014 and 2021 were superimposed and juxtaposed with the original line map from Wright *et al*. [18]. This was done to synchronize track numbering with that of Wright *et al*. [18] and ensure consistency in track placement, shape and surface exposure over time. The numbering system in figure 4 corresponds to that used in the individual photogrammetric models reposited on Zenodo.

**Figure 5.**
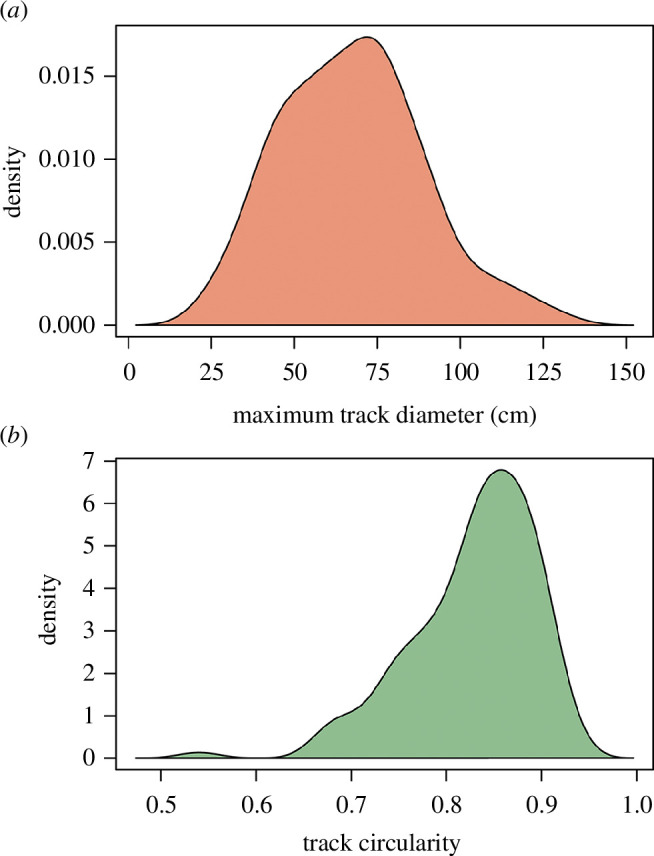
Histograms showing the distribution of track size (*a*) and track circularity (*b*) at Spyway Quarry.

We calculated the maximum diameter and circularity of individual tracks from the final summary scaled line model (figure 4) using the automated image measurement function in ImageJ [29] (figure 5 and electronic supplementary material, table S1). We note that the outline of the track is used incorporating the displacement rim in part because of difficulties consistently determining the exact boundaries of tracks owing to poor preservation. Thus, these outlines are assumed to be minor overestimates of the actual track size. Tracks that are not completely exposed because they are truncated by the edge of the quarry were excluded from the analysis.

Six blocks containing sauropod tracks, each weighing approximately 200−300 kg, were removed from Lewis Quarries as part of the initial conservation plan for the site developed by the National Trust. These blocks are stored outside at Keates Quarries Ltd and are currently accessible with the permission of Kevin Keates. The original and more extensive Lewis Quarries track surface was digitized in 2018 [25], but the model data generated during this work is not publicly accessible and was not available for examination as part of the current study. The site has now been quarried and is no longer accessible. Given the variable quality and completeness of the tracks exposed on the accessible blocks, we present selected three-dimensional models representing two of the blocks as per the photogrammetric methods described for Spyway Quarry.

## 4. Results

### 4.1. Spyway Quarry

Wright *et al*. [18] noted 111 tracks scattered across the bedding surface, with an additional three deemed indeterminate (tracks 1−114 in figure 4). Using the 2014 and 2021 photogrammetric models alongside field observations in 2021, a further 24 tracks were identified, bringing the total number of tracks at the site to 138 (tracks 115−138 in figure 4 [20]). These new tracks are primarily situated along the perimeter of the quarry in the northern, northwestern and eastern sectors, where a larger surface area was exposed in 2014 compared with the present day or that exposed in 1998. The reduction in track surface size since 2014 results from vegetation encroachment and collapse/creep of the embankments. Additionally, several tracks identified in 1998 were no longer visibly distinct on the surface in 2021, while others appeared faint, probably owing to continued erosion [20].

**Figure 6.**
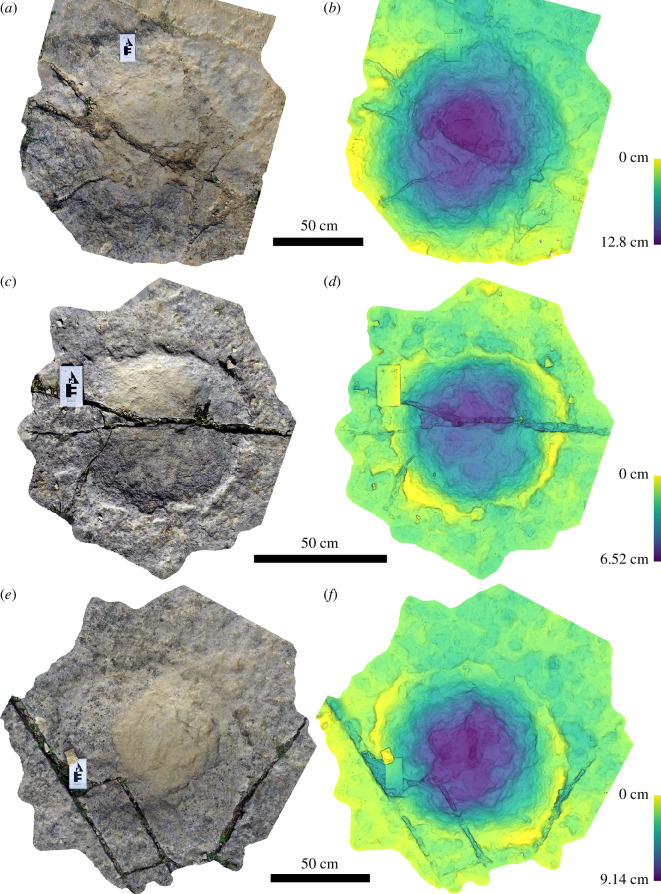
Typical ‘bowl-like’ sauropod pes impressions from Spyway Quarry, showing tracks 1 (*a, b*), 10 (*c, d*) and 28 (*e, f*). Tracks are shown as images of three-dimensional photogrammetric models (*a, c, e*) and depth maps (*b, d, f*).

Tracks are generally preserved as subcircular to oval depressions with low raised rims that rise 10−30 mm above the main trackway surface (figure 6). None of the tracks preserve good morphological details such as the impressions of claws or toes. As such, they should be considered poorly preserved (scoring 0 on the preservation scheme of Belvedere & Farlow [30]). We interpret the outlines of the tracks as probably not fully representative of the original morphology in many cases owing to visible erosion of original track rims. In general, there is no clear grouping of individual tracks into trackways, although there are three examples of aligned series of tracks of similar sizes that could potentially represent parts of trackways (tracks 18, 19 and 20; tracks 12, 13 and 14; tracks 103, 104, 111 and 135). The latter two of these sets of aligned tracks run parallel to one another, and it is possible that they could represent left and right tracks within a wide-gauge trackway.

**Figure 7.**
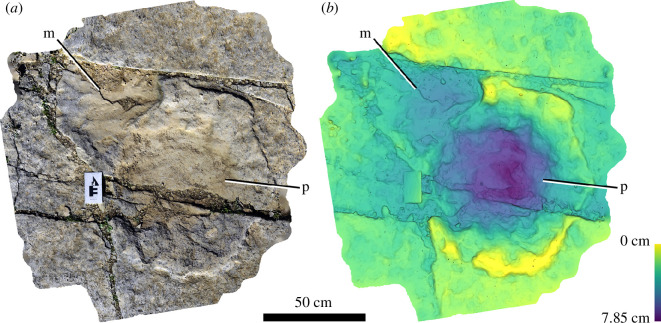
Manus (track 49) and pes (track 15) pair from Spyway Quarry. Tracks are shown as images of three-dimensional photogrammetric model (*a*) and depth map (*b*).

Tracks range from 24 to 130 cm in maximum diameter (electronic supplementary material, table S1) with a mean diameter of approximately 67 ± 22 (1σ) cm, with most between 50 and 90 cm (figure 5*a*). Tracks overall have a high mean circularity score of 0.83 ± 0.07 (1σ) (where 1 equals a perfect circle) appearing largely roughly circular to slightly elliptical in shape (figure 5*b*). The majority of tracks are most likely pes (i.e. rear foot) based on their shape. However, a small number of manus tracks are probably present, based primarily on an inferred D-shape outline (noting, however, that sauropod tracks interpreted as manus impressions can also appear quite circular in outline, e.g [31]). Several of these manus tracks are closely associated with pes prints. In Wright *et al*. [18], 14 manus prints were tentatively identified (tracks 13, 17, 21, 23, 27, 29, 34, 36, 37, 39, 42, 49, 59 and 81), of which 13 were described as D-shaped. From an evaluation of the current quarry surface and the 2014 images, we can only identify three of these as manus with reasonable confidence (track 49 paired with pes print 15; tracks 36 and 42; figure 7), with five further tracks possibly identifiable as manus (tracks 13, 17, 21, 27 and 59). The preservation of the site and highly fractured surface make identification of D-shaped tracks challenging.

**Figure 8.**
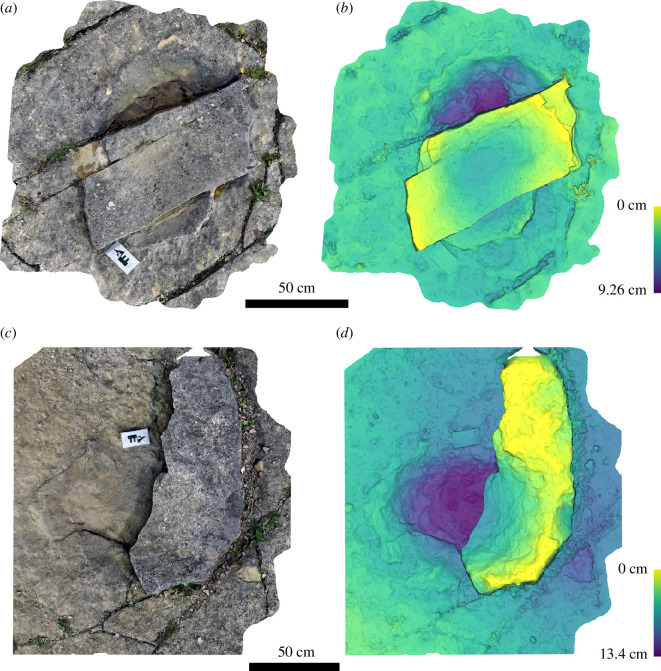
Tracks 61 (*a, b*) and 117 (*c, d*) showing that tracks are expressed in multiple layers within the sediment. Tracks are shown as images of three-dimensional photogrammetric models (*a, c*) and depth maps (*b, d*).

While the majority of the tracks appear on the surface of a single bedding plane, in tracks 4, 60, 61 and 117, there is evidence of overlying layers present across parts of the track evidencing that the same print and displacement rim occurs in multiple layers (figure 8). Sediment laminations drape the shape of the track and are roughly parallel to one another, including with well-defined displacement rims in places.

**Figure 9.**
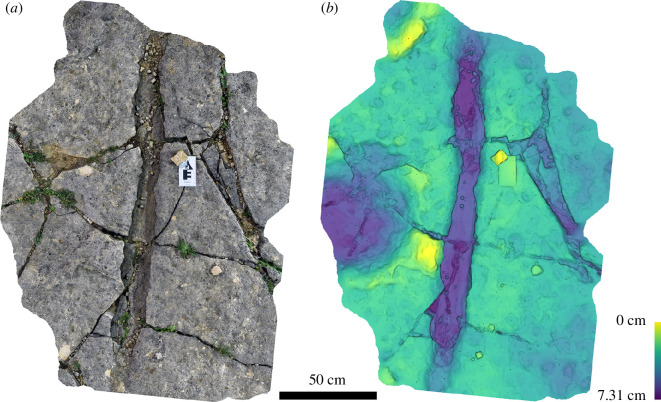
Groove on the main track surface previously interpreted as a tail drag but reinterpreted here as possibly representing an impression of fossil wood. The bowl-shaped depression adjacent to the groove is track 26. Groove shown as image of three-dimensional photogrammetric model (*a*) and depth map (*b*).

Other notable features observed on the bedding surface are two elongate grooves that taper at each end and are independent of the pervasive fracture network: the largest of these (approx. 3 m long and 9 cm wide) is adjacent to tracks 27 and 26 (figure 9) and the second is approximately 19 cm long and approximately 4 cm wide. In both cases, the impression extends below and is slightly overhung by a lip of the bedding surface. The smaller impression has roughly linear fine grooves exposed in its base. A number of hypotheses have been proposed to explain the large elongate groove on the quarry bedding surface including that it is a tail groove or foot drag mark [18]. Given the similarities of the two impressions plus the positions of the imprints relative to the tracks, we suggest that it is more likely that these represent impressions of pieces of wood, which are commonly preserved within the Purbeck Limestone Group (e.g. [32]).

### 4.2. Lewis Quarries

**Figure 10.**
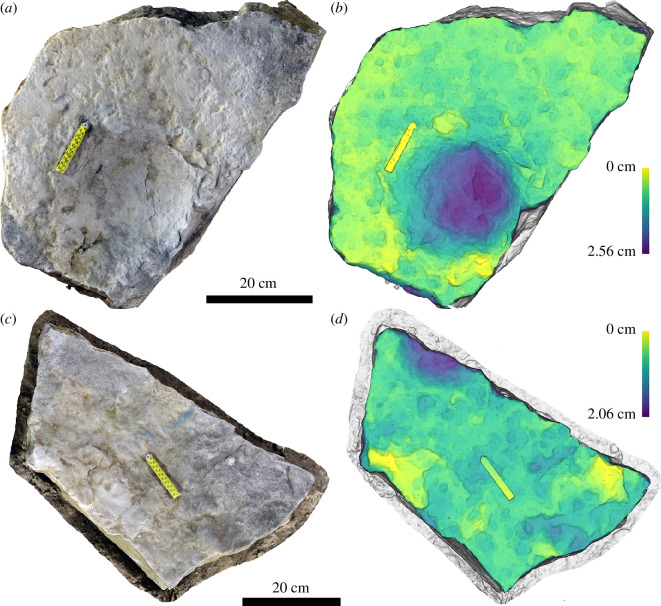
Images of selected track-bearing blocks from Lewis Quarries showing single sauropod tracks preserved as shallow, bowl-like depressions. In the lower block, the track is partial and preserved in the top left corner. Blocks are shown as images of three-dimensional photogrammetric models (*a, c*) and depth maps (*b, d*).

The six blocks each contain a complete or partial sauropod track (figure 10). Unfortunately, no track labels or connection to the original position of these on the bedding surface is available. The tracks are relatively similar to those at Spyway but more poorly preserved. Of those that appeared relatively complete, maximum diameter ranges from 54 to 82 cm including extensive displacement rims, with a smaller internal measurement of 38−47 cm. They are preserved as shallow sub-circular depressions (approx. 2 cm at the deepest point) on the top surface of the Bottom Freestone and lack any distinguishing features.

## 5. Discussion

### 5.1. True tracks versus transmitted tracks

Determining whether tracks are true tracks or transmitted undertracks is potentially important for interpretations of the trackmaker’s body size, behaviour, taphonomy and stratigraphic position. Transmitted tracks are often considered of lower scientific value than true tracks, i.e. those forming within the bed on which the dinosaurs directly walked, and the interpretation of the Spyway tracks as transmitted was part of the rationale for the opening of the site to the public without restricting access to the track surface [33]. The initial investigation of the site suggested that the tracks were true tracks [18]. Evidence for this was based on the identification of infilling sediment layers that drape the track and onlap to the edges of the track and sedimentary evidence from the raised rims (unknown track in fig. 7 of Wright *et al.* [18]). The lack of morphological detail preserved within the tracks was attributed to the sediment type and environmental conditions rather than track transmission, which would result in a progressive loss of detail with increasing depth in the sediment [34]. Subsequent unpublished interpretations [33] suggested a transmitted origin and that the original track surface was higher in the sequence. This question is difficult to answer with certainty for the Spyway tracks. We noted that evidence of tracks in layers overlying the main track surface occurs in several parts of the site, which provides suggestive but not conclusive evidence that the tracks on the main surface are transmitted. Regardless of the interpretation, we noted that the identification of the Spyway tracks as either true or transmitted does not necessarily limit the scientific significance of the site, depending on the questions posed.

### 5.2. Trackmaker identity

The very large size and sub-circular, bowl-like appearance of the pedal prints, the apparently D-shaped manus print (where preserved) and the earliest Cretaceous stratigraphic occurrence indicate that the tracks at Spyway and nearby Lewis Quarries were made by sauropods, the dominant group of large-bodied herbivorous dinosaurs in the Late Jurassic—earliest Cretaceous interval [8]. Fish-feeding traces can be preserved with a similar appearance to sauropod tracks (e.g. [35]); however, the preservation of well-developed rims and the placement of D-shaped manus tracks in close relationship to more bowl-shaped pes tracks makes this interpretation unlikely for the Spyway tracks. The apparent overall limited preservation of manus tracks is not necessarily unusual, and the occurrence of pes-dominated sauropod track sites has been suggested to relate to the position of the centre of mass of the trackmaker (e.g. [36]).

A more refined taxonomic identification than Sauropoda is hampered by the lack of unambiguous trackways, the limited number of manus-pes sets, and the generally poor preservation of the tracks. As a result, few of the synapomorphies of taxonomic groups within Sauropoda identified by Carrano & Wilson [37] as potentially preservable in tracks can be adequately assessed. One feature that would potentially be significant would be whether trackways have footprints that approach the midline (narrow gauge) or are widely separated from the midline (wide gauge), or are intermediate between the two (intermediate gauge) [37–39]. Narrow gauge trackways have been inferred to be ancestral for sauropods as a whole, with a transition to wide gauge trackways via intermediate gauge trackways proposed to occur within the clade Titanosauriformes (Brachiosauridae + Titanosauria) [37–39]. Early members of this group, such as brachiosaurids, are proposed to have the intermediate gauge track morphology. In two areas of the quarry, there are groups of tracks that might be interpreted as parts of trackways—if the two parallel aligned sets of tracks 12, 13 and 14 and tracks 103, 104, 111 and 135 were interpreted as left and right sides of a single trackway, then this would appear to be wide gauge. However, the identity of the aligned prints as trackways is difficult to confirm. Moreover, it has been documented that the gauge of sauropod tracks can be variable along a trackway dependent on variations in behaviour and substrate (e.g. [40]). As a result, the usefulness of this character for identifying the trackmaker at Spyway is limited.

Sauropod body fossils are extremely rare in the Purbeck Limestone Group [22]. Only isolated teeth and an incomplete metacarpal bone have been discovered, and both are currently identified as Sauropoda indet. [22]. The underlying Portland Group also yields only indeterminate sauropod material [22]. Elsewhere within the UK and Europe, Tithonian–Berriasian sauropod body fossils are also relatively infrequent, but most occurrences have been assigned to either turiasaurs or macronarians (including titanosauriforms) (e.g. [41–43]), although records of diplodocoids have also been reported (e.g. [44,45]). As such, there are three major groups of sauropods known from the Tithonian–Berriasian interval that could have produced the tracks at Spyway, and the poor preservation of the Spyway tracks does not help in distinguishing between these clades.

**Figure 11.**
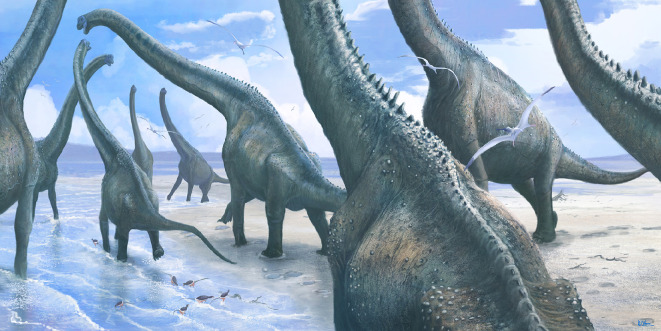
Life reconstruction of the wider environmental setting of the Bottom Freestone during the formation of the Spyway tracks showing large brachiosaurid sauropods crossing a lagoonal environment. Small heterodontosaurid dinosaurs and flying pterosaurs are shown based on body fossil occurrences elsewhere in the Purbeck Group, although there is no direct evidence for these taxa at Spyway. Artwork copyright Mark Witton.

Wright *et al*. [18] identified brachiosaurids (early titanosauriforms within Macronaria) as the most likely trackmaker for the Spyway tracks based on their relatively common body fossil occurrences in this interval and the large size of the tracks. Although this identification is plausible (figure 11), the limited information available for the Spyway tracks means that the trackmaker cannot be confirmed beyond Sauropoda.

### 5.3. Palaeobiological inferences

Assessment of the number of individuals represented at the site is complicated by the lack of definite trackways and poor preservation. Variation in the maximum dimensions of the prints appears to be normally distributed around a mean value of approximately 67 cm (figure 5), and there is no clear indication of a bimodal distribution or multiple peaks in the data. While the large range in maximum track dimensions may indicate more than one size class of trackmakers, some of this may be variations in size that can be present in transmitted tracks and/or erosion of the boundaries of the tracks (e.g. [34]). The large number of tracks at the site is consistent with the possibility of multiple individuals, although this inference is complicated by the lack of definite trackways. Sauropod trackways recovered elsewhere, including within strata of approximately equivalent age, have provided clear evidence of gregarious behaviour in the clade (e.g. [46,47]). The tracks at Spyway are consistent with such behaviour. They also provide evidence for sauropods inhabiting or at least passing through a coastal lagoonal setting, as also indicated by many previous sauropod track occurrences (e.g. [2,48,49]).

Sauropod hip height has been traditionally estimated from footprints via formulae that use pes length [50–52]. Using a mean track diameter of 67 cm, the equation of Alexander [50] would suggest a minimum hip height of approximately 2.7 m, although this might be a substantial underestimate given the considerably larger size of some of the tracks, which might suggest animals with hip heights up to 4−5 m. The minor overestimation of track dimensions introduced by the production of automated measurements from the track site map (figure 4) is unlikely to have a substantial impact on these size estimates.

### 5.4. Significance of the site

There are very few examples of sites in the UK that preserve multiple exposed sauropod tracks within a single horizon. Late Triassic trackways from Bendrick Rock in South Wales are of basal sauropodomorphs rather than true sauropods and are much smaller in size with a different morphology from those at Spyway [13,53]. Tracks within a nearby but stratigraphically younger Late Triassic horizon at Penarth probably represent sauropodomorph, and potentially sauropod, track makers, but their interpretation is somewhat controversial [4] and they are poorly preserved and variably exposed over time ([4]; RJB and KME 2023, personal observation). The originally described trackway surface in Middle Jurassic deposits at Ardley in Oxfordshire, which preserves multiple sauropod trackways [14–16], has been buried and is no longer accessible, although new exposures of tracks have occurred recently as a result of ongoing quarrying ([54]; RJB and KME 2023, 2024, personal observation). Unfortunately, three-dimensional models of the Ardley site are also not available as it was discovered and documented prior to the widespread use of three-dimensional scanning and photogrammetry approaches. Two sauropod track sites have been recently described in the Middle Jurassic of the Isle of Skye, Scotland [2,3].

Other sauropod trackways and isolated tracks have been reported previously from the Purbeck Limestone Group (e.g. [17,22,27,28]), although these are generally either isolated examples (e.g. [28]) or may in some cases represent other groups such as ankylosaurs [55]. As described above, the sauropod tracks from Lewis Quarries [25] are poorly preserved and the original associations of individual tracks relative to one another have been lost.

As such, Spyway represents the most important record of sauropod tracks from the Purbeck Limestone Group and the largest dinosaur track site within the Group that is currently accessible *in situ*. In terms of the number of individual tracks, it is also the largest readily accessible dinosaur track site in the UK (given that new exposures of tracks at Ardley Quarry are temporary and not publicly accessible) and thus of considerable scientific significance and popular interest. Although the site is deteriorating owing to exposure to the elements [20], it is likely that the rate of deterioration is slower than at most other UK dinosaur track sites, which are generally exposed on coastal sections. The stratigraphic position of the site in the earliest Cretaceous (Berriasian) is also important, as it documents the presence of large sauropods shortly after the Jurassic/Cretaceous boundary, which has been suggested by some as a possible interval of heightened extinction rates, particularly among sauropodomorphs [8].

## 6. Conclusions

Here, we have provided the first published scientific description of Spyway Quarry, which preserves more than 130 individual tracks of large sauropod dinosaurs from the earliest Cretaceous of Dorset. Morphological preservation of the tracks is poor and it is not possible to group tracks clearly into trackways, limiting the useful information that can be obtained from them. Despite their poor preservation, the site remains significant as sauropod tracks are rare in the UK, and Spyway is the largest *in situ* dinosaur track site currently accessible within the Purbeck Group. There is potential for future discoveries through ongoing quarrying in the surrounding area, which could yield specimens that are more informative in terms of trackmaker identification and behavioural inferences.

## Data Availability

Data for the 2021 model of Spyway quarry is available at [56]. All newly generated photogrammetric models have been uploaded to Zenodo [57]. Supplementary material is available online [58].
